# Engaging patients in health research: identifying research priorities through community town halls

**DOI:** 10.1186/s12913-017-2138-y

**Published:** 2017-03-11

**Authors:** Holly Etchegary, Lisa Bishop, Catherine Street, Kris Aubrey-Bassler, Dale Humphries, Lidewij Eva Vat, Brendan Barrett

**Affiliations:** 1 Clinical Epidemiology, Public engagement lead, NL SUPPORT, Faculty of Medicine, Memorial University, Craig L. Dobbin Centre for Genetics, Rm 4M210, St. John’s, NL A1B 3V6 Canada; 2 School of Pharmacy, Discipline of Family Medicine, Memorial University, St. John’s, Canada; 3 NL SUPPORT Unit, Faculty of Medicine Memorial University, St. John’s, Canada; 4 Primary Healthcare Research Unit, Discipline of Family Medicine, Memorial University, St. John’s, Canada; 5 Scientific Director NL SUPPORT Unit, Faculty of Medicine Memorial University, St. John’s, Canada

**Keywords:** Patient engagement, Research partners, Research priorities, Health, Town halls initiatives

## Abstract

**Background:**

The vision of Canada’s Strategy for Patient-Oriented Research is that patients be actively engaged as partners in health research. Support units have been created across Canada to build capacity in patient-oriented research and facilitate its conduct. This study aimed to explore patients’ health research priorities in the province of Newfoundland and Labrador (NL).

**Methods:**

Eight town halls were held with members of the general public in rural and urban settings across the province. Sessions were a hybrid information-consultation event, with key questions about health research priorities and outcomes guiding the discussion.

**Results:**

Sixty eight members of the public attended town hall sessions. A broad range of health experiences in the healthcare system were recounted. Key priorities for the public included access and availability of providers and services, disease prevention and health promotion, and follow-up support and community care. In discussing their health research priorities, participants spontaneously raised a broad range of suggestions for improving the healthcare system in our jurisdiction.

**Conclusions:**

Public research priorities and suggestions for improving the provision of healthcare provide valuable information to guide Support Units’ planning and priority-setting processes. A range of research areas were raised as priorities for patients that are likely comparable to other healthcare systems. These create a number of health research questions that would be in line with public priorities. Findings also provide lessons learned for others and add to the evidence base on patient engagement methods.

## Background

Engaged patients are research *partners*, involved in the planning, conduct and governance of health research, rather than serving as passive study subjects [1–3]. Patient engagement could improve the relevance of health research, facilitate the adoption of research findings into practice, and ultimately improve patient outcomes [1–7]. A continuum of engagement ranges from informing and consulting, to collaboration with researchers, to empowering, patient-led projects [8–10]. Patients can be engaged at all levels, including identifying study topics, collecting data or disseminating findings [1, 3, 9, 11].

In line with international patient engagement movements [11, 12], the Canadian Institutes of Health Research (CIHR) announced Canada’s Strategy for Patient-Oriented Research (SPOR) in 2011. Its vision is that “patients are active partners in health research that will lead to improved health outcomes and an enhanced health care system [13].” A key goal was the creation of Support for People and Patient-Oriented Research and Trials (SUPPORT) Units across the country whose mandate is to facilitate patient-engaged research on jurisdictional priorities.

The methods and impact of patient engagement are under-described [3]. Most studies provide little detail about engagement processes, few evaluate its impact over time, and uncertainty remains about best practices for supporting engagement [3, 9, 14]. Systematic reviews [3, 9, 15] reveal patient engagement is most common in the early stages of research, and a recent review highlighted how patient-identified priorities helped shaped research programs in Rheumatology, Venous Thromboembolism, and Paget’s disease [16]. However, research is often not aligned with the priorities of patients. For example, the James Lind Alliance analysed over 300 studies reporting research priorities and found that most described the priorities of researchers, not patients [17].

This article describes a patient engagement exercise at Memorial University in Newfoundland and Labrador (NL), Canada. A broad view of ‘patient’ was adopted, referring to any member of the general public who had experience with the healthcare system as a patient at some point in their lives. Common methods of engagement for exploring research priorities include meetings, workshops and focus groups [3, 16, 18]. This study used a town hall meeting format.

## Objectives

To explore health research priorities and outcomes of the public and to contribute to the evidence base on patient-oriented research.

## Methods

Approval was granted by the Newfoundland and Labrador Health Research Ethics Authority (Project Approval # 15.057).

### Planning sessions

The NL SUPPORT Unit partnered with a local SPOR research network, PRIIME (Primary Healthcare Research and Integration to Improve Health System Efficiency) to conduct town halls. Sessions were planned during monthly meetings in early 2015. The team was comprised of two clinicians, a pharmacist, and health researchers from a variety of areas (e.g., genetics, public health, primary care). All supported patient engagement in research and were committed to facilitating open, respectful discussion on patient-identified priorities and outcomes. Communication assistance was not offered for town halls, which could have precluded participation from some members of the public. No translation services were thought to be required as the population in this jurisdiction is largely homogenous (white, English-speaking, and middle class), and none was required.

### Town hall sessions

Town halls were a hybrid information-consultation session led by a team member with facilitation experience (HE). A 15–20 min presentation was developed, which introduced the idea of CIHR’s SPOR and provided an overview of the research initiative at Memorial. Discussion was centered around two questions to gather data on public priorities (Table 1). These were developed prior to sessions in order to facilitate discussion, but also to maintain consistency across town halls.

**Table 1:** Research questions used during town hall meetings

1. We are interested in what kind of health research projects are of interest to you. If you could think about a worry or concern about healthcare in this province that needs to be our top priority, what would that be?
2. We want to know what you would like to see happen as a result of health research. As a patient, what are the outcomes you might want to see as a result of health research?

At least two other team members attended each session. At the beginning of sessions, team members introduced themselves and explained their interest and endorsement of patient engagement with health research. In this way, an encouraging and supportive environment for conversation was created. Ground rules were explained following introductions, namely that no names were required or would be attached to any comments; we were interested only in summarizing participants’ ideas. We also asked that participants respect others’ views, acknowledging that everyone’s experience and priorities could be different. Participants were encouraged to share their views, even if these were different from those expressed and to give each other time to speak before commenting. The facilitator made every effort to encourage all participants to share their thoughts and moved conversation back to the key question if it became sidetracked with any one participant’s experience. This was generally done by asking if other participants had similar experiences or asking how this might be translated into a health research question or outcome.

### Recruitment

Sessions were planned in rural and urban settings. Posters were widely distributed to community organizations and posted in public locations (e.g., pharmacies, post office). Sessions were advertised on PRIIME and NL SUPPORT’s websites, and research team members extended invitations to their networks. Local call-in radio programs were used, as well as promotion by community organizations through websites and word-of-mouth.

### Data collection

Audio recordings of sessions were taken with two digital recorders placed at opposite ends of the room. A survey booklet containing the two questions above, as well as standard demographic items and three evaluation questions about the usefulness of a town hall format, was developed and provided to participants during the introduction of the session. Participants were encouraged to complete the demographic items and record any thoughts in response to the questions and conversation as the session progressed. Detailed notes, including quotes from participants, were taken by all members; flip charts were used to record key ideas, in particular health research areas identified by participants which were outlined in bullet form. Debriefing meetings were held where researchers’ observations and reflections about sessions were recorded. These meetings allowed researchers to review notes and discuss key areas of health research identified by participants as sessions progressed. This allowed a good understanding of issues raised in prior sessions and anticipatory reflection of what might be discussed at upcoming town halls (or easy identification of different ideas raised).

### Data analysis

Flip chart recordings, team members’ detailed notes, and data from completed survey booklets comprise the data for analysis. No pre-existing framework was developed in advance of the analysis; rather, an inductive approach was used to allow categories to emerge from the data. No qualitative software was used to manage the data. Qualitative description [19] was used to explore participant comments by HE and LB. This type of naturalistic inquiry made no *a priori* theoretical or philosophical assumptions about the data. The data are presented in the language of participants, resulting in a comprehensive summary of participants’ ideas.

## Results

Eight town halls sessions were held across the province with 68 members of the public (Table 2). Sessions lasted an average 1.5 h. Most participants did not complete survey booklets, preferring to engage in discussion. Thus, demographic information was not consistently captured. Observation indicated that participants represented a broad range of ages and backgrounds, with more females than males in attendance.

**Table 2:** Town hall session information

Town hall location	Date	Audience	Number attended
Church hall, St. John’s	April 2015	General public, mixed ages	21
Municipal office, Ferryland	June 2015	General public, mixed ages	4 (3 male)
Mall Walkers Club – senior’s group, St. John’s	October 2015	General public, most >65 years of age	15 (2 male)
Seniors Bridging Cultures – a senior’s group, St. John’s	November 2015	General public, >65 years of age	12 (4 male)
West Coast Tour, 4 communities Grand Falls-Windsor Deer Lake Corner Brook Port aux Basques	May 2016	General public, mixed ages	16 (4 male)

### Thematic analysis

All data elements were first read independently by HE and LB. Through an iterative process of reading the data, discussing, and rereading, these investigators consensually validated emerging categories. Data were compared between and within town hall sessions using constant comparative analysis [19, 20]. Constant comparison requires a constant shifting back and forth between data elements to establish analytical categories and themes, as well as their boundaries. For example, participants often talked about problems with accessing health services, making it a key theme. However, constant reading of the data revealed various foci of access, such as primary care and specialist services. Once HE and LB reached consensus, the summary of themes was presented to a subgroup of team members who had attended sessions for discussion. No new themes were identified.

The public’s priorities for health research are described under: 1) Access and availability of healthcare services; 2) Disease prevention, health promotion and healthy aging; 3) Survivorship, follow-up support and community care. Potential solutions spontaneously raised by participants are also described.

#### Access and availability of services

The most frequently raised priority for the public was access to healthcare services.

“I am here for two years and I am not able to find a physician.”

Even when people had a family physician, getting an appointment was not timely. “You have to make an appointment with your family doctor *before* you get sick.”

On-call services and after-hours access to family physicians, in particular to avoid unnecessary trips to the ER, were frequent concerns.

“I’d like to see my doctor when I’m sick and not have to go to the Emergency.”

“Better access to doctors results in reduced wait times… The problem is the necessity of using the emergency department for primary care. You need some sort of walk-in clinic if your physician is not available, and the clinic should have access to patient records.”

Enough time during appointments to discuss healthcare concerns was also raised. One senior noted,

“You feel like you are imposing on your doctor.”

Concerns about access extended well beyond access to family physicians. Frustration with excessive wait times for specialist appointments was evident. One participant noted it would be quicker to drive over an hour to another community’s ER with his child, than wait for an appointment with a pediatric specialist closer to his hometown. The difficulty of seeing a specialist was a shared concern at Seniors’ sessions. A breast cancer survivor told us:

“It’s terrible. If you have a problem, why should you have to wait six months to see someone?”

Very specific service concerns were noted. The lack of psychological support services, addiction services and general lack of mental health supports were apparent. A physician participant told us:

“I could run my whole practice on mental health issues for adolescents and young adults.”

Other specific areas of discussion included a lack of timely access to procedures such as MRI. In one community, the lack of assisted-living facilities for young adults with developmental disabilities such as autism was a concern.

Timely access to primary care providers, as well as a range of healthcare services and supports, was the most frequent concern of participants. In response, participants spontaneously discussed possible solutions, many of which were underscored by making better use of limited healthcare resources (e.g., having nurse practitioners provide primary care in communities, better use of telehealth in rural areas, a traveling clinic to provide basic screening services such as eye exams).

#### Disease prevention, health promotion and healthy aging

The need for better disease prevention and health promotion, rather than the current reactive nature of our healthcare system, was commonly noted. Seniors noted the importance of helping them stay at home for as long as possible, saving the health system money:

“This province has an aging population. If we can keep people healthy and not requiring excessive healthcare, this should free up resources for other matters.”

Participants in many sessions strongly endorsed the notion of health promotion at a young age. Discussion centered on the role of teaching health promotion and healthy living in schools as a way to reduce disease, as well as providing youth opportunities for physical activity.

While all participants recognized the value of health promotion, they noted that ongoing and early education required investment, both at the level of the educational system and at the level of patient engagement. There was recognition that patient engagement with health research might promote the idea of patients taking responsibility for their own healthy choices.

#### Survivorship, follow-up support and community care

Follow-up care and quality of life concerns were raised in nearly every town hall. Sometimes this was related specifically to cancer survivors’ quality of life, but also the health and well-being of their caregivers.

Several stories were related about movement through the healthcare system. These shared a common theme of feeling lost and isolated, and the discussion raised the importance of accessing follow-up care and support after treatment has ended:

“I’ve been in the system since [year]. But some things have fallen through the cracks. I almost died. I wished I had someone to talk to…you can get lost.”

There was general agreement that following treatment, “you are just left there out in the cold” and a recognition that family doctors do not have the time to provide needed emotional support. A cancer survivor reflected,

“Your family thinks it’s done [cancer], so they can’t understand why you would still be talking about it.”

Participants raised the issue of caregiver burnout and the need for research on home care following surgery or other treatment. Better support for informal caregivers was a perceived need as families are smaller and support networks are reduced. Participants suggested that incorporating family members in clinical care teams could provide better patient-centred care and help anticipate post-discharge needs.

#### Potential Solutions

A variety of solutions were raised by participants (Table 3), highlighting the breadth of public discussion.

**Table 3:** Potential solutions raised by town hall participants

Encourage prevention agenda – through better knowledge dissemination to patients, screening services that travel to rural communities, or teaching youth about healthy eating and physical activity Government investment in health promotion
Encourage patients to be more involved in their healthcare
Integrate family members into clinical care teams
Educate patients and physicians on how to talk to each other (not at each other)
Patient education on medications and the role of a pharmacist; patient education on the management of chronic conditions
Better use of pharmacists to do regular medication review with patients
Healthcare planning in rural areas of the province, not simply centering all services in the capital city; taking a long-term planning view (e.g., insulin pump rather than ongoing needles)
Appropriate use of acute care hospital beds
Appropriate use of transport (e.g., using a taxi instead of ambulance in non-emergency situations)
Appropriate use of healthcare professional time and expertise (e.g., nursing time is being spent on data entry and paperwork)
Expanding the role of healthcare professionals; some primary care service provision can be provided by nurses, midwives and pharmacists
Extending telehealth to other areas
Extending hours of home care available
Making it easier for patients to physically access healthcare facilities (e.g., better parking needed)
Integrate technology into healthcare (e.g., electronic medical records)
Ask patients about their experiences in an exit interview

## Discussion

Empirical description of engagement efforts is limited in the literature [3–7]. We used town halls to explore the public’s health research priorities and hope findings add to the evidence base and provide lessons learned for others engaged in patient oriented research.

In line with other research on patient priorities [7], timely and easy access to healthcare emerged as a key patient priority. With many rural settings and an ageing population of patients with multiple co-morbidities [20], it is unsurprising that access is a key issue in this jurisdiction. Findings concur with a recent survey of the provincial Medical Association that revealed most patients lack continuity of care with a family doctor due to high turnover rates, particularly in rural areas [21]. For those without a family doctor, 62% used emergency departments to receive primary care. However, majorities of patients reported being comfortable with receiving care from other providers such as nurses or pharmacists, and endorsed accessing their doctors through telehealth. Similar findings were noted in the provincial Premier’s Summit on Health Care, where coordination of care amongst health care providers was a key suggestion for better healthcare [22]. From a patient engagement perspective, these potential solutions create key research questions in line with public priorities.

Also in line with the public survey and health summit were discussions around the importance of promoting healthy eating and exercise in youth as a means of achieving long-term sustainability of the healthcare system [21, 22]. Childhood obesity and low activity levels are global health issues [23–25], and town hall participants noted the importance of introducing healthy living early in a child’s life. Reviews confirm the economic burden of obesity in Canada in part through increased usage of the healthcare system [25]. Participants stressed the importance of disease prevention and health promotion, and again, these areas provide researchable questions that can be considered when determining health research priorities.

Broadly, the public who attended town halls argued for a more efficient use of limited healthcare resources as a way to combat access problems and lack of follow-up care. They recognized that not every service could be provided in every community and acknowledged patients must play a role by taking responsibility for their health. Notably, topics raised by participants reflected areas of improvement needed, which themselves have been the focus of much research and quality improvement initiatives. Thus, perhaps the public discussions reflect a relative lack of implementation of available health research. Town hall discussions suggest a prioritization of health research designed to implement real change in the healthcare system.

Town halls posed some limitations. We do not know how representative the sample is of the provincial population. We did not consult with every area of the province, and community differences in care access and provision are likely. It is also possible that participants were already engaged with health in some capacity (e.g., healthcare professionals) or associated with a specific disease group. However, it is encouraging that study findings are similar to the results of a recent random digit dialling survey and health summit within the province [21, 22]. While we attempted to tape record sessions, large meeting spaces and many voices meant inaudible recordings. It is unlikely data saturation was reached, and additional sessions could have raised other priorities. Despite these limitations, this is the first study to explore public health research priorities in our province since the release of Canada’s SPOR strategy. Findings provide information to guide patient oriented research planning and to add to the evidence base on patient engagement methods. Town halls provided the opportunity and space for members of the public to discuss shared concerns about their health and healthcare. Patient knowledge includes a form of practical knowledge, a ‘knowing in action’ that is developed from daily living with chronic disease; it has been suggested this knowledge can be shared and made useful to others [26]. During town halls, participants often told stories about their journeys through the healthcare system or management of chronic disease that would be largely unexplored through standard survey items. Owing to their qualitative nature, town halls allowed rich descriptions of participant experiences, from which key research priorities and outcomes could be distilled. This required a skilled moderator, however, and we note other lessons learned below.

### Lessons learned

Checklists for patient engagement and lessons learned are emerging [11, 18, 27–29]. Many of these lessons are useful across a variety of engagement activities (e.g., thinking through the purpose of the engagement, ongoing communication with participants). Here, however, we provide specific lessons learned relating to using town halls as a way to solicit patient research priorities (Table 4).

**Table 4:** Town halls for patient engagement: Lessons learned

Lesson	Details
Language Matters	Public events were advertised with the word ‘research.’ As the purpose of town halls was to identify public research priorities, it seemed an obvious word choice. A participant at a town hall with a very low turnout explicitly advised the team that residents likely thought they could not contribute to a discussion about ‘research.’ He advised people simply be invited to attend a town hall about their health concerns. This advice is well taken, as a first hurdle is getting the public to come out. It would be instructive to involve patients in planning sessions and advertising plans.
Use a skilled facilitator at public sessions	Facilitators need to be able to paraphrase the public’s health concerns and distill these into research priorities and outcomes. An experienced facilitator will also steer the discussion from any one participant’s health concern or experience.
Use a variety of methods to engage the public	While town halls provide excellent opportunity for discussion and clarification, cold advertising resulted in low turnout that is not easily justified in terms of the resources needed. Practical matters such as trying to tape-record group discussion in large spaces also proved impossible. In recent months, we have found better reception when members of our team visit community groups directly. A key lesson is ‘go to them,’ rather than expecting the public to come to you.
Significant time is required for planning	Time to plan town halls must be accounted for in project planning and budgeting. Our teams met for up to six months to plan events before they started. Adequate time is needed for advertising upfront and for identifying appropriate community contacts to help advertise the event.
Determine how to describe the study sample	Town hall formats may not allow adequate description of the study sample given participants’ disinclination to complete booklets provided during the session. We recommend time at the beginning of the town hall to collect demographic information. Patient engagement activities could also require a re-thinking of standard participant descriptions in results sections of papers.

## Conclusions

Town halls can be a useful method to gather information about patients’ health research priorities. Through the conduction of town halls across the province, a broad range of health experiences in the healthcare system were raised, including access and availability of services, disease prevention and health promotion, and follow-up support and community care. Discussions came with many suggestions for improving the healthcare system. Public engagement proved to be valuable in guiding patient oriented research planning.

## Abbreviations

CIHR: Canadian institutes of health research
NL SUPPORT: Newfoundland and labrador support for people and patient-oriented research and trials
NL: Newfoundland and labrador
PRIIME: Primary healthcare research and integration to improve health system efficiency
SPOR: Strategy for Patient-oriented research
